# Hyperlipidemic microenvironment conditionates damage mechanisms in human chondrocytes by oxidative stress

**DOI:** 10.1186/s12944-017-0510-x

**Published:** 2017-06-12

**Authors:** Daniel Medina-Luna, Mónica Guadalupe Santamaría-Olmedo, Yessica Zamudio-Cuevas, Karina Martínez-Flores, Javier Fernández-Torres, Gabriela Angélica Martínez-Nava, Denise Clavijo-Cornejo, Cristina Hernández-Díaz, Anell Olivos-Meza, Luis Enrique Gomez-Quiroz, María Concepción Gutiérrez-Ruiz, Carlos Pineda, Francisco Blanco, Anthony M. Reginato, Alberto López-Reyes

**Affiliations:** 1 Synovial Fluid Laboratory, Instituto Nacional de Rehabilitación “Luis Guillermo Ibarra”, Calzada México Xochimilco 289, 14389 Mexico City, Mexico; 2 Departamento de Ciencias de la Salud, Universidad Autónoma Metropolitana Iztapalapa, Avenida San Rafael Atlixco 186, Iztapalapa, 09340 Mexico City, Mexico; 3 Musculoeskeletal and Articular Ultrasound Laboratory, Calzada Mexico-Xochimilco 289, Col. Arenal de Guadalupe, Tlalpan, 14389 Mexico D.F, Mexico; 4 Arthroscopy Service; Instituto Nacional de Rehabilitación “Luis Guillermo Ibarra Ibarra”, Secretaría de Salud, Calzada Mexico-Xochimilco 289, Col. Arenal de Guadalupe, Tlalpan, 14389 Mexico D.F, Mexico; 5 Rheumatology Division, ProteoRed/ISC III Proteomics Group, INBIC, A Coruña, Spain; 6 Division of Rheumatology, Warren Alpert School of Medicine at Brown University, Providence, RI USA

**Keywords:** Chondrocytes, Free fatty acids, Inflammation, Oxidative stress

## Abstract

**Background:**

Currently, two pathogenic pathways describe the role of obesity in osteoarthritis (OA); one through biomechanical stress, and the other by the contribution of systemic inflammation. The aim of this study was to evaluate the effect of free fatty acids (FFA) in human chondrocytes (HC) expression of proinflammatory factors and reactive oxygen species (ROS).

**Methods:**

HC were exposed to two different concentrations of FFA in order to evaluate the secretion of adipokines through cytokines immunoassays panel, quantify the protein secretion of FFA-treated chondrocytes, and fluorescent cytometry assays were performed to evaluate the reactive oxygen species (ROS) production.

**Results:**

HC injury was observed at 48 h of treatment with FFA. In the FFA-treated HC the production of reactive oxygen species such as superoxide radical, hydrogen peroxide_,_ and the reactive nitrogen species increased significantly in a at the two-dose tested (250 and 500 μM). In addition, we found an increase in the cytokine secretion of IL-6 and chemokine IL-8 in FFA-treated HC in comparison to the untreated HC.

**Conclusion:**

In our in vitro model of HC, a hyperlipidemia microenvironment induces an oxidative stress state that enhances the inflammatory process mediated by adipokines secretion in HC.

## Background

Osteoarthritis (OA) is a degenerative disease characterized by alterations in the cartilage, synovial membrane and subchondral bone that trigger the progressive and irreversible degeneration of the joint. OA is one of the most common musculoskeletal disorders of the aging population [1–3], and a leading cause of chronic disability. A great variety of risk factors contributes to the development of OA such as age, gender, joint trauma, genetic factors, excessive joint usage (such as physical cartilage-overload) and particularly obesity [4, 5]. Obesity is one of the most important risk factors for OA as the hyaline cartilage can be damaged in two different mechanisms: (1) biomechanical stress and (2) inflammatory systemic factors [6–8]. The first one is caused by an altered biomechanics (especially in the knee); whereas the second one is derived from a pro-inflammatory state regulated by the activation of adipocytes which are natural producers of adipokines: leptin, adiponectin, resistin and visfatin [9]. These proteins are systematically present, thus they are present also in the joint and could be involved in its degradation [4]; however, it is not clear if a highly lipidic microenvironment within the human cartilage may promote a similar response [10]. In OA patients, there are some proteins that play an important role in inflammation and degradation of the cartilage such as, metalloproteinase 9 (MMP9) and 13 (MMP 13), and cytokines such as interleukine-1 (IL-1), interleukine-8 (IL-8), interleukine-6 (IL-6), and Interferon-γ (IFN-γ). In addition, these molecules are also involved in the generation of nitric oxide (NO) and reactive oxygen species (ROS) such as superoxide anion (O_2_^·-^) and hydrogen peroxide (H_2_O_2_) [11–13]. The importance of ROS in OA is that these molecules are implicated in intracellular oxidation, especially in lipid peroxidation, which promotes cell membrane damage, protein oxidation and eventually lead to chondrocyte apoptosis [14–16]. An increase in ROS production in the joint is common in OA, and it has previously been reported that an imbalance in the chondrocyte metabolism could be related to an increase in chondrocyte lipid content [17].

The deposition of free fatty acids (FFA) within the joint stimulates both a deregulation of the metabolic process and an imbalance of the oxygen available. To address this issue, we used an in vitro HC model where we used FFA intra-cartilage as inductors of oxidative and inflammatory state.

## Methods

### Human articular chondrocytes

Hyaline cartilage biopsies, macroscopically healthy and  4 mm in diameter, were harvested by arthroscopy from a non-weight bearing area of the knees (lateral wall of the notch), from four consecutive patients who underwent surgery for Anterior Cruciate Ligament (ACL) repair. Three of these patients were female whereas one was male, with an average age of 44.25 ± 24.81 years, and a mean body mass index of 26.97 ± 5.33 kg/m^2^. All participants were appropriately informed about the study aims and signed an informed consent form. This study meets all the criteria contained in the Declaration of Helsinki and was approved by the Ethics and Research Committee of the Instituto Nacional de Rehabilitación (INR) (Ref. INR-08/11).

### Primary isolation and culture of human chondrocytes

**Fig. 1:**
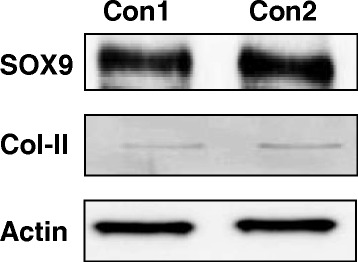
Phenotypic characterization of human chondrocytes: Western blot analysis of the transcriptional factor SOX 9, Collagen type II, and actin expressed by human chondrocytes in monolayer culture

Immediately after the harvesting process, the cartilage tissue was subjected to mechano-enzymatic breakdown in order to isolate the HC as previously described [18]. Cells were cultured in DMEM/F12 medium supplemented with 10% fetal bovine serum (FBS) and 1% antibiotics (Gibco, Thermo-Fischer Scientific, Waltham, MA USA). Cell cultures were maintained at 37 °C, with 5% of CO_2,_ at saturation humidity. Chondrocytes from the fourth passage were used for all experiments, the HC were characterized using Western Blot analysis for SOX9 transcription factor to ensure the phenotype of the cells were maintained all along the study (Fig. 1).

### Experimental design

To assure statistical significance each experiment was performed in triplicate for all patients (*n* = 4). HC were treated with a FFA mixture composed of palmitic acid (C16), and oleic acid (C18:1, cis-9) (Sigma-Aldrich St. Louis, Missouri USA), in a ratio 1:2 respectively at concentrations of 250 and 500 μM for 48 h, respectively. As a positive control for oxidative stress, 100 μM of H_2_O_2_ was used, and cells maintained under the same condition, while HC not exposed to FFA or H_2_O_2_ were used as a negative control. The lipid overload within the HC was confirmed by Oil red staining [19]. After the exposition to the FFA, the supernatant from cell cultures was collected and stored immediately at −20 °C, and cells were carefully harvested for the measurement of ROS and reactive nitrogen species (RNS).

### Oxidative stress measurement

ROS and RNS production was evaluated through intracellular determination of O_2_^.-^, H_2_O_2_ and NO using the Tali Image-based Cytometer (Life Technologies). O_2_^.-^ and H_2_O_2_ were quantified, by oxidation of the dihydroethidium (DHE) and the 5-, 6- carboxy-2′, 2′, 7′-dichlorofluorescein diacetate (carboxy- H2DCFDA) respectively (kit Image-iT LIVE Green Reactive Oxygen Species Detection). NO was detected using the commercial kit DAF-FM (4-amino-5-methylamino-2,7-difluorofluorescein diacetate, Molecular Probes). DHE was quantified in the 530 ± 20 nm emission filter, and the carboxy-H2DCFDA and DAF-FM fluorescence in the 458 ± 20 nm emission filters, according to the manufacturer’s instructions.

### Pro-inflammatory proteins quantification in HC culture medium

Pro-inflammatory cytokines were evaluated in the supernatant from FFA-untreated, FFA-treated, and H_2_O_2_ treated HC cells by Milliplex Human Adipocyte Magnetic Panel (Merck-Millipore, Darmstadt, Germany). The pro-inflammatory cytokine quantification was performed by Magpix Merck-Millipore and the results were expressed in ng/ml. The samples were analyzed by triplicate according to manufacturer’s instructions.

### Statistical analysis

Data are presented as mean ± standard deviation (SD). Statistical differences between experimental groups were assessed by one-way ANOVA test adjusted by Bonferroni multiple-comparison test. The mean of the estimated difference in ROS intracellular production relative units, as well as in secreted proteins concentration between groups was assessed by linear regression models. To assess the association between ROS intracellular production and secreted protein concentrations, Spearman correlation coefficients were calculated with their respective *P*-values adjusted by Bonferroni test combining data from both FFA concentrations. All *P*-values lower than 0.05 were considered as statistically significant. Prism v6.01 (GraphPad Software Inc., California, USA) and STATA v12.1 (StataCorporation, College Station, TX, USA) were used to perform the analysis and graphs.

## Results

### HC overload lipids under FFA treatment

**Fig. 2:**
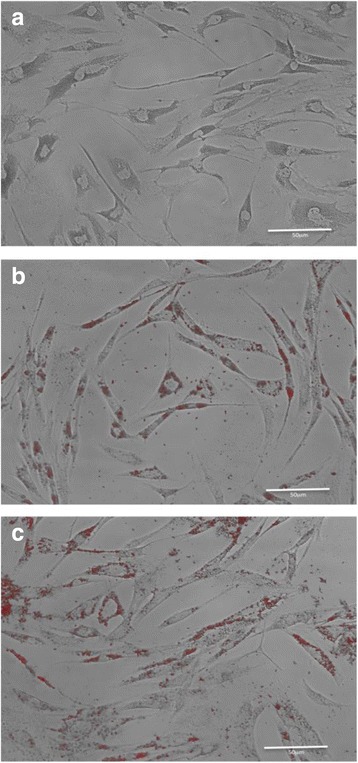
Free Fatty Acid Internalization: Oil Red O Staining of human chondrocytes (HC) exposed to 250 μM and 500 μM FFA. Free fatty acids induce the increment in the lipid content in human chondrocytes (HC) **a** Not FFA- treated HC. **b** HC exposed to 250 μM FFA and **c** HC exposed to 500 μM FFA for 48 hs. Representative images of three different monolayer cultures

Treatment of HC with 250 or 500 μM of FFA for 48 h lead to increase HC lipid overload by Oil red staining. Figure 2 shows that HC without FFA stimulation did not present significant lipid load (Fig. 2a); however, FFA-treatment, induced an overload of neutral lipids in exposed HC, suggesting an efficient cellular load at both FFA concentrations (Fig. 2b and c).

### FFA increase ROS and RNS production in HC

**Fig. 3:**
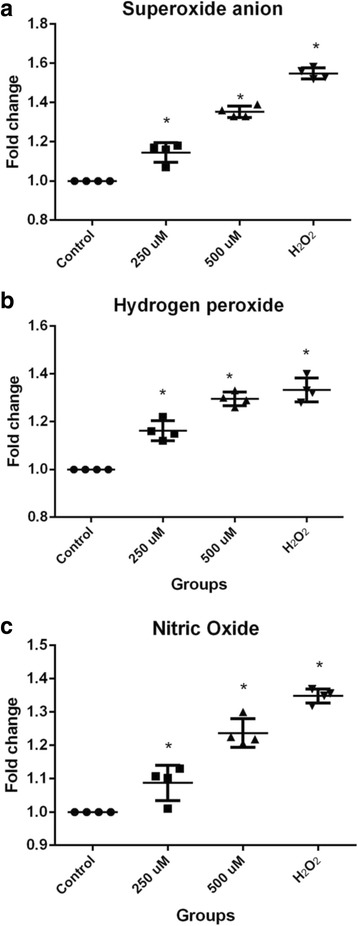
The intracellular content of free radicals: O_2_, H_2_O_2,_ and NO production by HC exposed to FFA. **a** O_2_^.-^. **b** H_2_O_2_. and **c** NO in HC treated with or without 250 or 500 μM of FFA, and H_2_O_2_ (100 μM) was used as positive control for 48 h. Each *bar* represents the mean value ± standard deviation of at least three independent experiments for each four different patients. ^*^ *P* < 0.05 with respect to untreated HC

HC treated with 250 and 500 μM of FFA for 48 h increased the production of ROS and RNS. The intracellular content of O_2_^.-^ increased to 14% in HC exposed to 250 μM of FFA (1.14 ± 0.05 vs 1.0, *P* < 0.01) and 35% when exposed to 500 μM of FFA (1.35 ± 0.02 vs 1.0, *P <* 0.01) compared to untreated HC (Fig. 3a). In addition, HC exposed to FFA showed an increase in H_2_O_2_ production of 17% when treated with 250 μM of FFA (1.17 ± 0.04 vs 1.0, *P* < 0.01), and 30% when treated with 500 μM (1.30 ± 0.03 vs 1.0, *P* < 0.01), compared to untreated HC (Fig. 3b). Similarly, the NO production increased to 11% (1.11 ± 0.02 vs 1.0, *P* < 0.01) and 24% (1.24 ± 0.04 vs 1.0, *P* < 0.01) in HC exposed to 250 and 500 μM of FFA, respectively, compared to untreated HC. (Fig. 3b). Our positive H_2_O_2_ control showed an increase of 56% (1.56 ± 0.05 vs 1.0, *P* < 0.01) in the O_2_^.-^ concentration, an increase of 34% (1.34 ± 0.07 vs 1.0, *P* < 0.01) in the H_2_O_2_ production and an increase of 35% (1.35 ± 0.02 vs 1.0, *P* < 0.01) in the NO production. (Fig. 3a-c).

### Free fatty acids induce pro-inflammatory secretion in HC

**Fig. 4:**
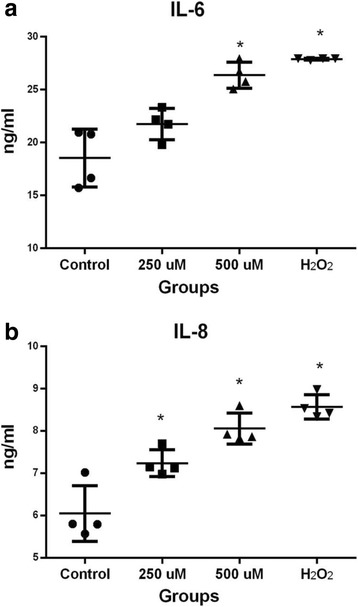
Quantification of cytokines: IL-6 and chemokine IL-8 in HC exposed to FFA. **a** IL-6. **b** IL-8 in HC treated with 250 and 500 μM of FFA, and H_2_O_2_ (100 μM) was used as positive control for 48 h. Each *bar* represents the mean value ± standard deviation of at least three independent experiments for each four different patients. ^*^ *P* < 0.05 with respect to untreated HC

In order to address the secretion of pro-inflammatory mediators elicited by lipid overload in HC, we determined the concentration of IL-6, and IL-8 in the supernatant of HC exposed to 250 and 500 μM of FFA for 48 h. IL-6 increased in a dose-dependent manner, having an average concentration of 21.7 ng/ml (*P* = 0.02) and 26.3 ng/ml (*P* < 0.01) with 250 and 500 μM FFA, respectively in comparison to 18.5 ng/ml in the untreated HC. In a similar fashion, the secretion of IL-8 increased from 6.1 ng/ml (untreated HC) to a concentration of 7.29 ng/ml (*P* < 0.01) and 8.11 ng/ml (*P* < 0.01) in the two concentrations of FFA treated HC (Fig. 4). For our positive control H_2_O_2,_ the concentration of IL-6 was 27.4 ng/ml (*P* < 0.01), for IL-8, the concentration was 8.6 ng/ml *P* < 0.01).

**Fig. 5:**
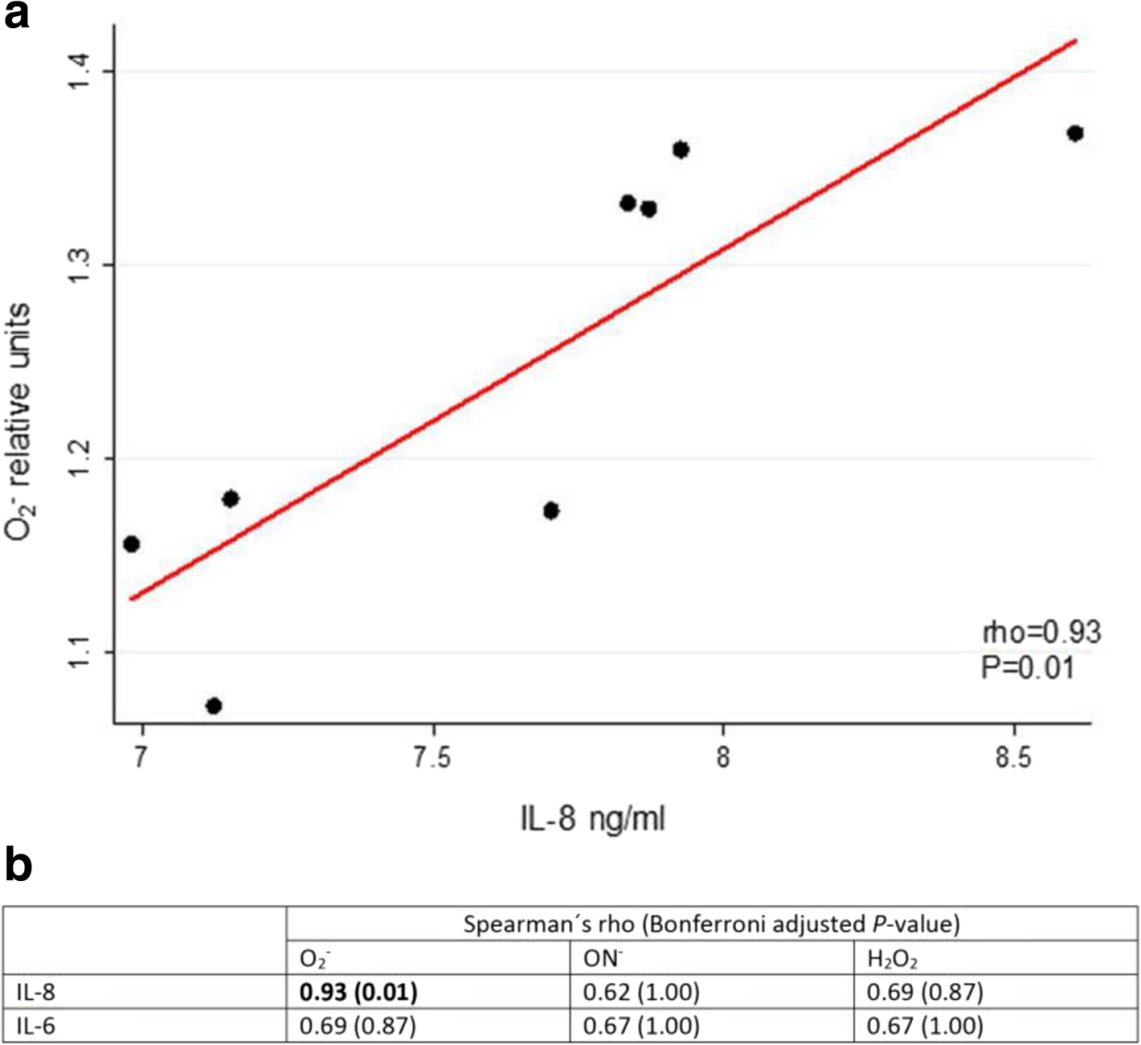
Statistical correlation between the results: **a** Positive correlation between O_2_^.-^ intracellular production and IL-8 concentration. **b** Spearman correlation coefficients ROS production and IL-6 and IL-8

A statistically significant positive correlation was identified between O_2_^.-^ intracellular production and IL-8 concentration (Fig. 5a) (rho = 0.93; Bonferroni-adjusted *P*-value = 0.01). Although, there was a positive correlation between NO and H_2_O_2_ production and IL-8 concentration, the magnitude was not statistically significant (rho = 0.62, *P* = 1.00; and rho = 0.69, *P* = 0.87). None of the IL-6 concentration and ROS production correlations were statistically significant, however, this data showed similar trend as IL-8 (Fig. 5b).

## Discussion

In this study, we observed that a lipidic microenvironment in human chondrocytes induces oxidative stress and elicits a proinflammatory response, that could be reflected in joint diseases. Our results show that FFA stimulates the production of ROS and RNS such as O_2_^.-^, H_2_O_2_, and NO, respectively, as well as the production of the cytokine IL-6 and chemokine IL-8 at the two FFA doses tested.

The role of ROS in the development of OA has been well documented. Henrotin et al. [15] showed that ROS promote chondrocyte apoptosis and inhibits matrix synthesis, therefore, promoting its breakdown [20]. In addition, several studies suggest that NO production is increased in chondrocytes from OA patients due to an overexpression of the inducible NO synthase (iNOS) causing chondrocyte apoptosis [21–23]. In an in vitro model, Sasaki et al. [24] found that NO has a deleterious effect in chondrocytes as it promotes the release of basic fibroblast growth factor (bFGF), which triggers the expression of metalloproteinases and iNOS. The NO could induce the production of cellular mediators that carry proteolytic properties on the extracellular matrix proteins such as fibronectin, type II collagen and hyaluronic acid [24]. The relevance of NO is highlighted by its signaling action, activating key signaling pathways in chondrocytes leading to in proteoglycan degradation, but also to the fact that it can induce the release of O_2_^.-^ by a donor compound to form peroxynitrite, creating a deleterious state in the cartilage as described by Scher et al. [20].

In our HC in-vitro model, we observed that at 48 h, the internalization of FFA by chondrocytes induces the production of ROS and RNS, suggesting that a hyperlipidemic microenvironment plays a key role in situ oxidative stress-state related to cartilage injury, characteristic in the OA development. In addition to the ROS production, we show evidence that HC stimulated with FFA leads to an increase on the cytokine IL-6 and chemokine IL-8 in  an autocrine fashion. IL-6 has been strongly associated with the development of OA as it acts as a mediator in the degeneration of the cartilage [25, 26]; furthermore, high levels of IL-6 have been associated with an increased loss of tibial cartilage [27]. Our results strongly suggest that FFA works as an inductor of cartilage damaged driven by oxidative stress that enhances the production of pro-inflammatory cytokines leading to cartilage damage.

On the other hand, IL-8 chemokine has also been shown to have a deleterious effect in OA [28]. IL-8 functions as neutrophils chemoattract at the injury site; increasing the content of ROS and others mediators of cell damage [29]. Moreover, ROS can induce IL-8 production along other inflammatory mediators such as prostaglandins that could lead to further tissue damage [30].

We provide evidence that the increase oxidative stress caused by HC exposed to FFA leads to ROS and RNS production, autocrine activation of IL-6 and IL-8 by chondrocytes leading to cartilage damage. Our data suggest that the oxidative stress along with an inflammatory response triggers an imbalance in the cartilage homeostasis as seen in OA patients with obesity [30]. Taken together, our data suggest that hyaline cartilage intra-substance with increased fatty acids, could induce an inflammatory response mediated by IL-6, IL-8, and oxidative stress in which free radicals, mainly NO, trigger the degeneration of the extracellular matrix. The highly lipidic microenvironment causes a cartilage damage by not only and increase in IL-1 [31], but also by an increase in ROS and RNS that enhances the autocrine expression of the cytokine IL-6 and chemokine IL-8 respectively. Our results provide a novel mechanism of increase oxidative stress in cartilage injury by FFA.

## Conclusions

**Fig. 6:**
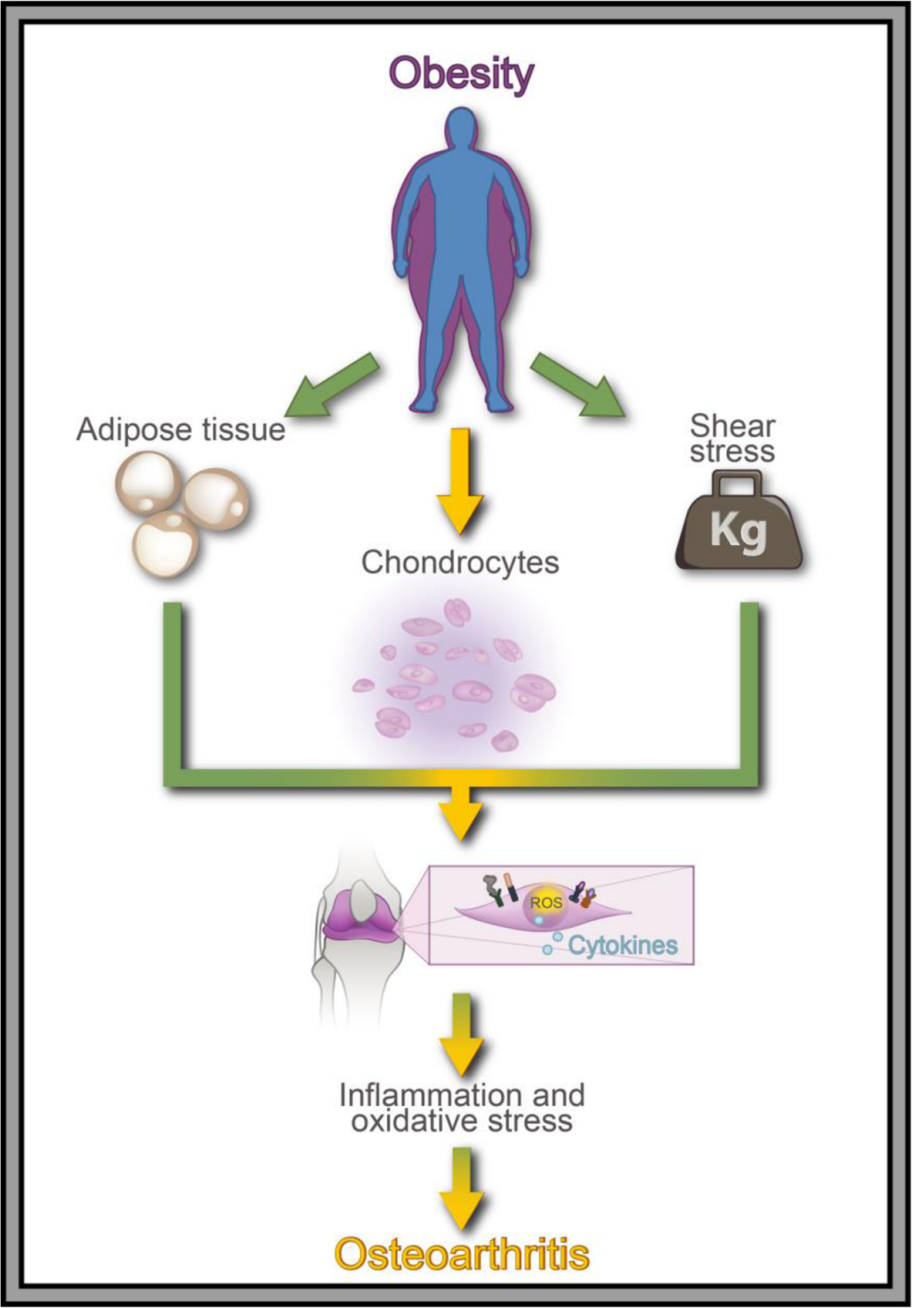
Graphic representation of the role of obesity: On oxidative stress leading to cytokine and chemokine autocrine production in OA. In this figure, it is possible to appreciate that the relation between obesity and OA is more than biomechanical stress where an increase in lipid composition in the cartilage induces the formation of pro-inflammatory mediators, ROS and RNS leading to cartilage damage

In conclusion, this work suggests an alternative mechanism in which an increase in the lipid composition in the cartilage also contributes to the development of OA and may suggest why non-weight-bearing joints such as the small joints of the hand are affected in OA patients with obesity and dyslipidemia (Fig. 6). Although our results are robust, further studies to the specific effect of FFA over HC all of these to elucidate more clearly the relation between obesity and osteoarthritis, considering the complex interaction of genetic, metabolic and biomechanical factors.

## Abbreviations

FFA: Free fatty acids
HC: Human chondrocytes
IL-6: Interleukin-6
IL-8: Interleukin-8
OA: Osteoarthritis
RNS: Reactive nitrogen species
ROS: Reactive oxygen species
